# Mind‐Muscle‐Environment Interactions: Psychophysiological Determinants of Optimal Pacing in Olympic Winter Endurance Sports

**DOI:** 10.1111/sms.70215

**Published:** 2026-02-10

**Authors:** Peter Edholm, Lars J. L. Bouten, Hans‐Christer Holmberg, Thomas Losnegard, Florentina Johanna Hettinga

**Affiliations:** ^1^ Department of Environmental and Biosciences, School of Business, Innovation and Sustainability Halmstad University Halmstad Sweden; ^2^ School of Health Sciences Örebro University Örebro Sweden; ^3^ Department of Human Movement Sciences VU University Amsterdam Amsterdam the Netherlands; ^4^ Division of Machine Elements Luleå University of Technology Luleå Sweden; ^5^ School of Kinesiology University of British Columbia Vancouver Canada; ^6^ Department of Physiology and Pharmacology, Biomedicum C5 Karolinska Institutet Stockholm Sweden; ^7^ Department of Physical Performance The Norwegian School of Sport Sciences Oslo Norway; ^8^ Norwegian Olympic Federation Oslo Norway

**Keywords:** biathlon, cross‐country skiing, endurance performance, pacing, psychophysiology, speed skating, winter sports

## Abstract

Pacing is a critical determinant of performance in Olympic endurance winter sports such as cross‐country skiing, biathlon, and speed skating. Although the physiological (“engine”) and psychological (“operator”) determinants of pacing have frequently been examined in isolation, growing evidence highlights the need for an integrated psychophysiological perspective. This structured narrative review first outlines the defining characteristics of Olympic winter sports and, within this context, synthesizes current knowledge on the psychophysiological mechanisms governing pacing to inform optimal preparation strategies. We describe how the physiological engine, encompassing energy systems, fatigue, and afferent feedback, and the psychological operator, involving self‐regulation, expectations, and cognitive‐affective processes, interact to shape pacing behavior. Moreover, the distinctive demands of winter endurance events necessitate consideration of environmental factors such as head‐to‐head racing formats, variable weather conditions, and undulating terrain. The affordance competition hypothesis is proposed as a unifying framework for understanding pacing as a continuous decision‐making process driven by dynamic mind‐muscle‐environment interactions. Finally, practical recommendations for athletes and coaches preparing for the Milano–Cortina 2026 Winter Olympic Games are presented, advocating a phased training approach that integrates physiological and psychological development within ecologically valid environments. Future research should prioritize real‐world competition settings, neurocognitive mechanisms, and underrepresented populations, including women and para‐athletes. We conclude that Olympic success in modern endurance winter sports depends on mastering an integrated psychophysiological control system, where performance is determined by both physical capacity and its effective regulation under varying environmental conditions.

## 1 Introduction

Cross‐country skiing, biathlon, and speed skating are cornerstone disciplines in the upcoming Milano–Cortina 2026 Olympic Winter Games, accounting for nearly one‐third of all medal events [1]. These sports impose physiological, psychological, and tactical demands, shaped by high‐intensity efforts, diverse race formats, and technical complexity. The outdoor disciplines of cross‐country skiing and biathlon are further challenged by variable environmental conditions, such as temperature, snow quality, wind, and altitude, which can significantly impact performance outcomes [2, 3, 4, 5]. In these disciplines, the ability to pace effectively and adapt to varying environmental conditions becomes a crucial factor in achieving competitive success.

The demanding nature of these sports, characterized by progressive fatigue in both the individual and mass‐start races, necessitates highly adaptive pacing skills and strategies. Both cross‐country skiing and biathlon are contested on undulating terrain, requiring frequent transitions between sub‐techniques. This results in repeated high‐intensity fluctuations, a pattern described as a “stochastic intensity profile”, which requires continuous regulation of energy expenditure [6]. In cross‐country skiing, athletes must dynamically adjust their sub‐technique (balancing upper‐, lower‐, and whole‐body work), energy distribution, and tactical positioning in response to speed variations caused by changes in terrain, snow conditions, wind, and competitor interactions [7]. Biathlon adds another layer of complexity by requiring athletes to perform a fine motor task, precision rifle shooting, under extreme physiological and psychological stress [8].

Speed skaters experience high demands due to sustained isometric forces during the crouched position. This posture, coupled with the rapid concentric force generation required during lateral push‐off at high velocities, severely restricts blood flow and leads to elevated metabolic stress and profound muscular fatigue [9, 10, 11]. Due to asymmetrical intramuscular forces when skating around corners, the duration and patterns of muscle re‐ and de‐oxygenation differ between long‐track and short‐track speed skating. As a result, short‐track skating is generally more physiologically demanding and associated with greater perceived fatigue and longer recovery times [11]. These fatigue‐driven processes not only impair physical performance but likely influence pacing decisions by altering perceived effort and pain sensations [11]. Subsequently, in both field (short‐track skating) and laboratory (cycling) settings, inter‐relationships between fatigue, responses to opponents and pacing have been established [12, 13].

From a physiological perspective, effective pacing reflects the interplay between aerobic and anaerobic energy systems, fatigue resistance, thermoregulatory control, and neuromuscular coordination. Athletes must optimize energy distribution and preserve mechanical efficiency across varying race demands [14, 15]. At the same time, psychological factors, including motivation, emotional regulation, perception of effort, decision‐making under stress, and responding to changing environmental conditions, play a decisive role in shaping real‐time pacing behavior [14, 16]. Despite their combined importance, physiological and psychological determinants of pacing have often been studied in isolation [17].

However, an emerging body of research advocates taking a psychophysiological perspective [17], where pacing emerges from the continuous and integrated interaction between mind and body, and also human–environment interactions play a central role. The first framework to systematically include environmental influences as impacting pacing decisions was the affordance competition hypothesis applied to pacing [16], based on an ecological approach [18]. Within this framework, pacing is not viewed as a fixed plan to cover a known distance from A to B as fast as possible, but as an adaptive, moment‐to‐moment decision‐making process governed by both internal cues and external demands, which allows adaptations based on environmental circumstances.

Previous reviews of pacing have typically examined single sports, often implicitly assuming time‐trial situations, and only in the last decade has the focus expanded to competitive events where environmental factors are more influential [17, 19]. The current work aims to synthesize evidence from Olympic winter endurance sports, cross‐country skiing, biathlon, and speed skating, which, despite differing environments, all involve varying external demands such as terrain (for skiing), ice and weather conditions, and direct interactions with competitors in repeated heats or rounds. These factors highlight the importance of environmental influences, which will be examined within a unified psychophysiological framework. We move beyond traditional pacing models by considering novel theories that conceptualize pacing as an adaptive, real‐time decision‐making process influenced by opponent behavior, terrain, and environmental conditions.

Although Milano–Cortina 2026 serves as a timely case example, the pacing framework presented here is intended to extend well beyond the upcoming Games. By integrating physiological, psychological, environmental, and decision‐making factors, it provides a durable structure applicable to future Olympics, World Cup seasons, and athlete development. The 2026 Games offer an illustrative context, but the underlying concepts are broadly relevant to pacing research and performance support across winter sports.

We aim to integrate the latest psychophysiological evidence and apply these insights to the uniquely interactive and stochastic demands of these three Olympic endurance winter sports. By adopting the affordance competition hypothesis as an overarching framework, this review offers a new perspective on how interactions between mind, muscle, and environment shape pacing in these Olympic winter sports, providing timely implications for athletes and coaches preparing for Milano–Cortina 2026.

## 2 Methods

This study is a structured narrative review, and the methodology was guided by the Scale for the Assessment of Narrative Review Articles (SANRA) criteria.

### 2.1 Search Strategy and Selection Criteria

A systematic search of three electronic databases (PubMed/MEDLINE, Web of Science, and Scopus) was conducted from their inception to Sep 2025. The search strategy combined three concept blocks: (i) the sporting disciplines (cross‐country skiing, biathlon, speed skating), (ii) pacing and effort regulation, and (iii) psychophysiology and decision‐making. To ensure comprehensive coverage, backwards reference screening and forward citation tracking of included articles were also performed. Studies were eligible if they were peer‐reviewed, published in English, and investigated physiological/physical, psychological/behaviouristic, or combined psychophysiological mechanisms of pacing or effort regulation in elite (national‐world class) athletes in the target sports. Studies focusing on youth, para‐athletes, or recreational cohorts, or those without direct relevance to pacing were excluded.

### 2.2 Screening, Data Charting, and Quality Appraisal

The selection process was conducted in Excel, where two reviewers [PE, HCH] independently screened all titles and abstracts, followed by a full‐text review. Disagreements were resolved by discussion within the research group. Following selection, key data were charted using a piloted form to capture study design, for example, sample characteristics, environmental conditions, pacing metrics (e.g., speed distribution, surges, and drafting), psychophysiological variables (e.g., rate of perceived exertion (RPE), heart rate (HR), lactate, and neuromuscular function), and principal findings.

### 2.3 Narrative Synthesis

Given the heterogeneity in study design, we synthesized the findings thematically rather than performing a meta‐analysis. Instead, the findings were thematically and narratively synthesized. The results were structured across four key themes: (1) Study design, (2) Sample size, sex and competition level, (3) perspective on pacing, including physiological/physical, psychological/behaviouristic and psychophysiological, and (4) main findings regarding pacing. Papers included are presented in Table 1.

**TABLE 1:** Summary of studies on pacing in Olympic endurance winter sports, detailing design, participants, research perspective, and main findings.

Sports discipline	Author/Year	Study design	Sample size (*N*)	Competition level	Perspective on pacing			Main findings
					Phy.	Psy.	Psy‐Phy.	
Cross‐country skiing (skating sprint)	Andersson, et al., (2010) [20].	Experimental	9	Elite	Yes	No	No	Sprint performance was largely determined by uphill sections performance; high VO_2_max, greater use of Gear 3 and higher double poling/Gear3 Velocity max related to faster times.
Cross‐country skiing (sprint)	Andersson, et al., (2016) [21].	Experimental	10	Well‐trained	Yes	No	No	Skiers employ positive pacing, incorporating high‐intensity surges uphill. Physiological strain and pacing are influenced by terrain.
Cross‐country skiing (sprint)	Andersson, et al., (2019) [22].	Experimental	34 (14 F)	Elite	Yes	No	No	Sex difference in time‐trial performance was ca. 12%, with male skiers relatively faster, especially on uphill sections, where they used G3 more frequently. Speed profile and power output were highly variable for both sexes, while HR variation was low.
Cross‐country skiing (skiathlon)	Fabre, et al., (2015) [23].	Experimental	6	National	Yes	Yes	No	During a skiathlon, the transition from classical skiing and skating alters performance. Skating + Skating yield lower aerobic energy expenditure, RPE and neuromuscular function.
Cross‐country skiing	Falk Neto, et al., (2024) [24].	Review	N/A	All	Yes	No	No	Skiers adopted variable pacing strategies, adjusting to terrain changes, environmental conditions, and race dynamics.
Cross‐country skiing (10 km skating)	Formenti, et al., (2015) [25].	Experimental	11	Highly Trained	Yes	No	No	Skiers used a reverse J‐shaped pacing with very high intensity (≈67% of time > 90% HRmax); slight end‐spurt; speed dipped mid‐race; HR rose progressively.
Cross‐country skiing (sprint)	Haugnes, et al., (2019) [26].	Experimental	12	Elite	Yes	Yes	No	A conservative start yields better sprint performance and lower fatigue in classic but not skate sprint when compared with a fast start.
Cross‐country skiing (sprint)	Haugnes, et al., (2022) [27].	Experimental	30	Elite	Yes	No	No	Tactical pacing is critical as advancement from heat to finals was linked to being in top positions before the last decisive uphill before the finish, and the ability to ski fast in that segment.
Cross‐country skiing (sprint)	Haugnes, et al., (2023) [28].	Observational	60 (30 F)	Elite	Yes	No	No	Throughout the heats, there is a gradual increase in the importance of being positioned at the front, in which the majority of performance variance was decided before the start of the finish sprint, both in men and women, in the classical and skating styles.
Cross‐country skiing (sprint)	Hébert‐Losier, et al., (2016) [29].	Review	567 (ca. 26% F)	Elite	Yes	Yes	No	Successful pacing tactics in sprint races involve the ability to attain high speed at the start of the race, when challenged by an opponent and at the final section of each heat, despite fatigue. Strong uphill performance is especially important.
Cross‐country skiing	Holmberg, et al., (2015) [7].	Review	N/A	Elite	Yes	No	No	Emphasizes terrain‐dependent intensity distribution (hard uphills, recover on downhills) and the ability to rapidly change between sup‐techniques/gears depending on terrain and skiing speed.
Cross‐country skiing (sprint)	Ihalainen, et al., (2020) [30].	Observational	11 F	Elite	Yes	No	No	Micro‐pacing in uphill and flat sections was strongly linked to total race time, with the best skiers pacing conservatively early and pushing harder on the final uphill. Effective micro‐pacing during transitions, uphill to flat and flat or uphill to downhill, helps sustain acceleration and maximize speed before tucking into a crouch position.
Cross‐country skiing (10/15 km ind. start)	Losnegard, et al., (2016) [31].	Observational	36 (14 F)	Elite	Yes	No	No	Both sexes used positive pacing; slower‐ranked men, but not women, started relatively faster and slowed more, suggesting that slower‐ranked men could benefit from more even pacing.
Cross‐country skiing	Losnegard, et al., (2019) [32].	Review	N/A	Elite	Yes	No	No	Overall positive pacing, but varying across terrain and speed to optimize performance. At high speeds on flat terrain, more power is lost to air drag, and because energy turnover is lower in double poling than in diagonal stride, these factors influence pacing across different terrain.
Cross‐country skiing (7.5 km ind. start)	Losnegard, et al., (2022) [33].	Experimental	34 (18 F)	Highly trained jr	Yes	Yes	Yes	In fast starters, a more even pacing strategy reduced early HR and RPE and improved time‐trial performance.
Cross‐country skiing (sprint)	Losnegard, et al., (2024) [34].	Experimental	16 F	Highly trained jr	Yes	No	No	High‐performance athletes performed better in a time‐trial sprint race using a fast start vs. a conservative start, while lower performers did not. A fast start was associated with higher discomfort in both high and lower performers.
Cross‐country skiing	Losnegard, et al., (2025) [6].	Review	N/A	Elite	Yes	Yes	Yes	Emphasizes the interplay between physiology, psychology, and tactics in shaping pacing, highlighting future directions for training. Pacing strategies should be individualized.
Cross‐country skiing (10 km ind. start)	Marsland, et al., (2017) [35].	Observational	8	National	Yes	No	No	Continuous macro‐kinematic data show that skiers pace by adjusting velocity, cycle rate, and technique across terrain. Effective pacing relies on terrain‐specific sub‐technique use and optimized cycle timing.
Cross‐country skiing (sprint)	Marsland, et al., (2020) [36].	Experimental	6 F	Elite	Yes	No	No	Skiers adjust pacing and technique across course sections based on terrain, tactics, and personal strengths. Tracking macro‐kinematic data helps tailor training to optimize pacing and performance.
Cross‐country skiing	Ni, et al., (2022) [37].	Simulation	N/A	Elite/National	Yes	No	No	Optimization pacing strategy reduced simulated skiing time; the best strategy included conserving power in the first uphill while increasing power in the latter half of uphill sections.
Cross‐country skiing	Sandbakk, et al., (2017) [38].	Review	N/A	Elite	Yes	No	No	Terrain‐dependent intensity distribution and high aerobic/anaerobic capacity are key to optimal pacing and success in skiing. A positive pacing strategy is commonly used.
Cross‐country skiing (sprint)	Sandbakk, et al., (2011) [39].	Observational	16	Elite/National	Yes	No	No	Elite skiers pace races through strong aerobic capacity, efficiency, and quick recovery, allowing sustained speed and repeated high‐intensity efforts. Their training enables aggressive starts and consistent speed across heats.
Cross‐country skiing (sprint)	Sandbakk, et al., (2011) [40].	Observational	12	Elite	Yes	No	No	Top skiers pace by sustaining speed on flat and uphill terrain, especially late in the race. Their success relies on maintaining high speed, efficient technique, and longer cycles to optimize performance.
Cross‐country skiing	Sandbakk, et al., (2014) [41].	Review	N/A	Elite	Yes	No	No	Optimal pacing in skiing depends on terrain, snow, and altitude, requiring skiers to balance effort across varying conditions. In mass‐starts, drafting and positioning are key for efficient pacing
Cross‐country skiing (10 km individual start)	Sandbakk, et al., (2016) [42].	Experimental	10 F	Elite	Yes	No	No	All skiers used a positive pacing strategy, but performance was mainly determined by technique‐specific aerobic power. Uphill performance and VO_2_ capacity in key techniques were the strongest predictors of success.
Cross‐country skiing	Sandbakk, et al., (2016) [43].	Experimental	12	Elite & National	Yes	No	No	Elite skiers pace effectively by maintaining rapid yet long double‐poling cycles, enabling high speed and sustained power. Their ability to preserve cycle rate under fatigue is key to consistent pacing and performance.
Cross‐country skiing (10 km individual start)	Seeberg, et al., (2022) [44].	Experimental	26	National/Well‐trained	Yes	No	No	Terrain‐specific micro‐pacing training helped skiers reduce time on flat and downhill sections, showing improved efficiency, though it did not enhance overall race performance.
Cross‐country skiing (21.8 km, mass‐start)	Seeberg, et al., (2022) [45].	Experimental	Ca. 57	Elite/National	Yes	Yes	Yes	Effective pacing in mass‐start skiing requires managing intensity shifts, maintaining uphill speed, and timing a strong final sprint, supported by smart positioning and race awareness.
Cross‐country skiing (10 km ind. start)	Shang, et al., (2022) [46].	Experimental	14	Elite/National	Yes	Yes	No	Skiing performance was associated with consistent pacing and effective use of skating sub‐techniques, especially in uphill segments. G3 and G2 were the most used sub‐techniques.
Cross‐country skiing (sprint)	Shang, et al., (2024) [47].	Experimental	14	National	Yes	Yes	Yes	Faster skiers gained most time on steep uphills by using longer cycles and more G3 technique, showing that effective pacing and technique adaptation on climbs are key to sprint performance.
Cross‐country skiing (10/15 km, ind. start)	Solli, et al., (2020) [48].	Observational	33 (8 F)	Elite	Yes	No	No	Men paced differently by using more double poling and less diagonal stride than women, reflecting both higher speed and distinct pacing strategies independent of terrain or speed.
Cross‐country skiing	Solli, et al., (2024) [49].	Review	N/A	Elite	Yes	Yes	Yes	Women tended to show steadier pacing profiles than men, while men more often employed variable strategies and aggressive surges.
Cross‐country skiing (4.3 & 13.1 km ind. start)	Sollie, et al., (2021) [50].	Experimental	19	National/Highly trained	Yes	No	No	Young skiers show higher intensity and a stronger positive pacing pattern than trained adults, indicating that pacing and technique training should be tailored to age and skill level.
Cross‐country skiing (10 km ind. start)	Staunton, et al., (2022) [51].	Experimental	19	Elite	Yes	No	No	Faster skiers maintained higher speeds on uphills and flats, showing distinct micro‐pacing differences between men and women. Mapping analyses help tailor training and optimize pacing strategies.
Cross‐country skiing	Stöggl, et al., (2018) [2].	Review	N/A	Elite	Yes	Yes	Yes	Skiers can enhance pacing by maintaining long, efficient cycles and improving endurance and strength. Less experienced skiers should use an even pacing strategy instead of starting too fast.
Cross‐country skiing (10 and 15 km ind. start)	Stöggl, et al., (2018) [52].	Observational	82 (41 F)	Elite/National	Yes	No	No	Pacing differs by sex and terrain: men gain most on flat sections, while women excel on steep uphills. Optimal pacing involves efficient technique use with fewer transitions and more double poling.
Cross‐country skiing (15 km ind. start)	Vikestad, et al., (2022) [53].	Observational	400 starts	Elite/National	Yes	No	No	Lower‐level skiers tend to start too fast and slow more over laps, while stronger athletes pace more evenly and sustain higher speeds, especially on uphills.
Cross‐country skiing (15 km ind. start)	Welde, et al., (2017) [54].	Observational	36	Elite/National	Yes	No	No	In a 15 km race, speed and cycle length declined over laps, most on flats and least uphill, showing terrain‐specific pacing and technique differences between faster and slower skiers.
Biathlon (sprint and individual)	Björklund, et al., (2022) [55].	Observational	Ca. 8600 starts (Ca. 50% F)	Elite	Yes	No	Yes	Top athletes maintained higher speeds throughout the race, especially within the final lap. Pacing interacts with shooting bouts and penalties.
Biathlon (pursuit and mass‐start)	Björklund, et al., (2022) [56].	Observational	Ca. 8600 starts (Ca. 50% F)	Elite	Yes	No	No	Faster skiing in the fourth lap is linked to better biathlon results and associated with ranking. Pacing helps but shooting accuracy has a much greater impact on performance.
Biathlon	Laaksonen et al. (2018) [8].	Review	N/A	Elite	Yes	Yes	Yes	Pacing is shaped by the need to balance high skiing speed with shooting accuracy; physiological strain impacts shooting precision.
Biathlon (distance, individual sprint)	Losnegard et al. (2023) [57].	Experimental	38 (11 F)	Highly trained	Yes	Yes	Yes	A fast start improved skiing time without harming shooting accuracy when compared to a more conservative start.
Biathlon (sprint)	Luchsinger et al. (2018) [58].	Observational	1800 starts	Elite	Yes	No	No	The top‐10 skiers had more even pacing. Most skiers used a J‐shaped pacing with a fast start and an even faster final lap vs. mid laps. No sex differences in pacing strategies.
Biathlon (distance, individual start)	Luchsinger et al. (2019) [59].	Observational	592 starts	Elite	Yes	No	No	The top‐10 skiers had a more even pacing. Most skiers used a J‐shaped pacing with a fast start and an even faster final lap vs. mid laps. No sex difference in pacing strategies
Biathlon (distance, pursuit)	Luchsinger et al. (2020) [60].	Observational	240 (128 F)	Elite	Yes	No	Yes	Athletes starting early used a more conservative start compared to those starting later in the pursuit race, who started more aggressively.
Biathlon	Staunton et al. (2024) [61].	Experimental	20 (7 F)	Highly trained	Yes	No	No	Performance‐critical segments were identified in both uphill and downhill sections. Rifle carriage shifted the importance to the uphill sections.
Speed skating (1500 m)	de Koning et al., (2005) [62].	Experimental + Simulation	8 (2 F)	Elite	Yes	No	No	The simulated “power balance model” accurately predicts performance and supports pacing optimization in speed skating.
Speed skating (1500 m)	Hettinga et al., (2011) [63].	Experimental + Simulation	7	National	Yes	No	Yes	Theoretical “optimal simulated pacing” does not always yield the best real‐world performance due to technique degradation.
Speed skating (Long‐ and Short‐track)	Hettinga et al., (2016) [11].	Experimental	12 (1 F)	Elite	Yes	Yes	No	Pacing in speed skating differs by discipline: short‐track requires sustained effort due to prolonged right‐leg loading and slower reoxygenation, making it more physiologically demanding than the more balanced long‐track style.
Speed skating (short‐track)	Hext et al., (2022) [64].	Observational	6196 starts	Elite	Yes	No	No	Race‐specific athlete–opponent interactions significantly enhance the analysis of pacing and tactical positioning in short‐track speed skating.
Speed skating (long‐track)	Konings et al., (2015) [10].	Review	Ca. 2100 starts	Elite, trained	Yes	Yes	Yes	Multiple person‐related characteristics interact to influence pacing and performance, also dependent on the race distance to determine which characteristics are more important.
Speed skating (short‐track 1500 m)	Konings et al., (2016) [65].	Observational	510 starts	Elite	Yes	No	No	Tactical positioning at one of the foremost positions during the latter phase of the race is a strong determinant of finishing position.
Speed Skating (short‐track)	Konings et al., (2018) [13].	Observational	410 starts	Elite	Yes	No	No	Races the same day, but not races the day before, impact pacing behavior in the upcoming race.
Speed skating (short‐track 500–1500 m)	Koning et al. (2018) [66].	Observational	30.094 starts	Elite	Yes	No	No	Competitive environments modify pacing, especially early in the race, highlighting how athlete–environment interactions critically shape behavior during head‐to‐head competition.
Speed skating (long‐track; > 3000 m)	Muehlbauer et al., (2010) [67].	Observational	Ca. 480 starts (Ca 50% F)	Elite	Yes	No	No	In long‐distance skating, a positive pacing strategy is sex, the athlete's level or venue altitude.
Speed skating (long‐track 1500 m)	Muehlbauer et al., (2010) [68].	Observational	114 (53 F)	Elite	Yes	No	No	Short sector time during the latter (not early) race segments is predictive of a short total race time in the 1500‐m middle‐distance event. A fast start may impede an optimal speed later in the race.
Speed Skating (1000 m)	Muehlbauer et al., (2010) [69].	Observational	65 (34 F)	Elite	Yes	No	No	Skaters used a fast‐start strategy, but final performance relied on faster closing laps. Lower‐ranked skaters should therefore train to sustain speed rather than maximize early acceleration.
Speed skating (short‐track, 500–1000 m)	Noorbergen et al., (2016) [70].	Observational	457 races (229 F)	Elite	Yes	No	No	Performance in short‐track sprint distances is primarily shaped by tactical behavior and positioning.
Speed skating (long‐track: mass‐start)	Peng et al., (2022) [71].	Observational	601 (251 F)	Elite	Yes	No	No	In the mass‐start, the pacing behavior differs by race, laps, skater level, competition stage, and sex, where top‐level skaters show more efficient pacing patterns.
Speed skating (long‐track: 1500 m)	Stoter et al., (2016) [72].	Experimental	9	National	Yes	No	No	During the final laps of 1500 m skating, fatigue negatively affects technique and pacing behavior. Fast starters had a greater loss in skating velocity compared to slow starters in the final laps.
Speed skating (team pursuit)	van den Brandt et al., (2023) [73].	Experimental	18 (9 F)	Elite	Yes	Yes	Yes	In the team pursuit, drafting reduces heart rate and perceived exertion. However, interindividual differences in physical and technical skills highlight the need for tailored pacing strategies.
Speed skating	van Schenau et al., (1990) [74].	Simulation	5	Elite	Yes	No	No	For the 500–1000 m, a fast initial acceleration is crucial for final performance. For the longer distances, constant power output is more important.
Speed skating (sprint)	van Schenau et al., (1994) [75].	Review	N/A	Elite	Yes	No	No	Sprint performance is optimized with fast‐start pacing strategies across modalities. Even pacing should only be used in events longer than 80 s.

## 3 The Pacing Puzzle: Foundational Concepts in Winter Endurance Sports

Pacing is a multifactorial, goal‐directed process through which athletes regulate exercise intensity over a known duration to maximize performance while avoiding premature fatigue or catastrophic failure [17, 76, 77]. Effort distribution can follow broad archetypes such as positive, negative, even, all‐out, or variable pacing strategies [14]. Early physiological models attributed performance limitation primarily to peripheral failure [78], but this view has been progressively replaced by psychophysiological frameworks recognizing the anticipatory and regulatory role of the brain in modulating performance [19, 79, 80, 81]. Models such as *teleoanticipation* [82], the *Central Governor* [83], and Marcora's *psychobiological model* [84, 85] each conceptualized endurance performance as the balance between physiological capacity, task knowledge, and the conscious or subconscious regulation of effort.

Across these frameworks, pacing emerges as a learned skill shaped by interaction between afferent feedback, feedforward control, prior experience, task and endpoint knowledge, and motivational state [76, 86]. This learning process relies on and, as such, is a skill that can be learned through self‐regulation processes [81, 87], and can be visualized through the development of a “performance template” [88]. In winter endurance sports, this regulatory challenge is further complicated by environmental variability (e.g., temperature, wind, altitude, and snow conditions), equipment–athlete interaction (e.g., ski‐snow friction, aerodynamic positioning), and opponent behavior [2, 66, 89]. For instance, altitude exposure can create a mismatch between internal physiological signals and external performance outputs, requiring a recalibration in pacing strategies [90]. Consequently, the performance is continuously refined through a self‐regulatory cycle of planning, monitoring, and evaluating responses to changing demands to optimize future performances [81, 88].

### 3.1 The Engine: Physiological Determinants of Pacing

From a physiological perspective, the athlete operates a complex “engine” in which pacing serves as the control system, balancing performance drive within physiological limits. Continuous afferent input from metaboreceptors, mechanoreceptors, nociceptors, thermoreceptors, and baroreceptors informs the central nervous system (CNS) about the current homeostatic state [79]. In teleoanticipatory models, athletes integrate prior experience and anticipated race demands with ongoing sensory feedback to ensure completion within physiological limits [77, 80, 82, 88]. One hallmark of this regulation is the deliberate constraint of motor‐unit recruitment: even during efforts perceived as maximal, a functional muscle reserve remains to safeguard homeostasis [79, 91, 92]. However, in supra‐maximal time‐trial settings, where athletes can self‐regulate effort to avoid premature task failure, declines in power output toward the end of a middle‐distance race are not accompanied by reductions in iEMG activity [93]. This suggests that peripheral fatigue, rather than central inhibition, is the primary driver of performance decline in such contexts.

Pacing is further shaped by the interplay between energy system dynamics and external resistive forces. In events lasting ~2 min on flat, windless terrain (e.g., indoor long‐track speed skating), an even power output is typically most metabolically efficient as it minimizes the energetic cost of repeated accelerations [63, 94]. In speed skating, however, technical and postural demands affect aerodynamic drag and mechanical efficiency [72], contributing to the common use of a positive pacing strategy characterized by a fast start [69]. For undulating terrain, characteristic of cross‐country skiing and biathlon, mathematical modeling proposes that variable pacing, with increased effort on climbs and reduced effort on descents, yields superior performance compared with an even pace, despite incurring a higher overall metabolic cost [37]. These terrain‐dependent changes in resistance (e.g., slope, aerodynamic load, ski‐snow friction) require athletes to modulate power output accordingly [2, 3]. World‐class cross‐country skiers exemplify this by generating small oxygen deficits on uphill sections and recovering on descents, resulting in a characteristic “stochastic interval profile” [2, 6]. This strategy is supported by modeling studies in cycling, where increasing work rate in headwinds or uphill and reducing it in tailwinds or downhill shortens overall time for the same mean power [95, 96]. Nevertheless, excessive power output on uphills may lead to fatigue that impairs downhill skiing technique, resulting in performance losses [29].

Beyond duration, terrain, and athlete interactions, event format also imposes key tactical constraints that shape pacing in winter sports. In individual‐start events, athletes primarily pace themselves based on internal cues and the demands of the course. In contrast, mass‐start races introduce drafting, responses to competitors' surges, positioning tactics, and risk–reward decisions. These dynamics can produce effective but physiologically “unorthodox” pacing patterns, such as prolonged high‐intensity starts or repeated surges. Such behaviors reflect both athlete‐specific strengths, for example, strong aerobic endurance enabling front‐leading strategies and the need to continually adapt pacing to situational demands [45, 56, 71].

### 3.2 The Operator: Psychological Determinants of Pacing

If the physiological systems form the athlete's “engine”, the psychological systems act as the “operator”, the set of cognitive and motivational processes that determine how the engine is driven. These systems include mechanisms that regulate attention, motivation, emotional responses, expectations, and effort [81, 97]. Together, they shape how athletes interpret internal signals and translate them into pacing decisions [81, 88]. Within this regulatory process, the Rating of Perceived Exertion (RPE) functions as a central control signal guiding adjustments in effort, integrating current sensations with expectations about task demands [84, 98]. Expectations and beliefs can shift this interpretation: deception studies, for instance, demonstrate that when athletes underestimate a task, they can access deeper physiological resources and exceed performances achieved under accurate conditions [99]. Such findings indicate that pacing decisions are not dictated solely by afferent input but also by the brain's interpretation of those signals within a broader cognitive and motivational context [16, 76].

Behavioral factors add another important layer of influence. Opponents, teammates, and race importance (e.g., World Cup race vs. Olympic game race) provide external cues or circumstances that can override strategies optimized for purely physiological efficiency [17, 89]. In head‐to‐head competitive formats, athletes often deviate from physiologically optimal time‐trial pacing to achieve the physiological benefits of drafting [73], respond to surges, or manage the psychological demands of the competitive situation [17, 19]. Here, the opponent becomes a dynamic external stimulus, continually shaping the athlete's pacing through an ongoing interplay between internal physiological state and psychological processing [73, 100]. In team competition events such as the team pursuit in long track speed skating, drafting takes an even more tactical and central role that is still understudied [73].

Operator engagement is also influenced by event format. In individual‐start races, operator input may focus on optimal distribution of effort and external feedback on split times, whereas in mass‐start events, operators must monitor positioning, group dynamics, and tactical triggers in real time. The frequency and nature of operator decision‐making can therefore vary markedly across race formats and even across phases within a single race (e.g., early positioning laps vs. decisive attacks). Recognizing these tactical layers is essential for translating pacing insights into effective performance strategies [16, 76].

### 3.3 The Mind‐Muscle‐Environment Interaction: An Affordance Competition Perspective

Contemporary perspectives emphasize that the athlete's physiological “engine” and psychological “operator” function as a tightly coupled, closed‐loop control system. Within this review, the ecological affordance competition framework is used to conceptualize pacing as a continuous process in which multiple potential actions (e.g., maintain pace, attacking, drafting) compete for selection within the central nervous system, that is, a flexible, adaptive system allowing room for responding to changing environmental circumstances [16, 18, 19]. In this view, decisions arise from the interplay between perceived affordances, opportunities for action defined by environmental conditions, task demands, and opponent behavior, and the athlete's internal physiological (“the engine”; e.g., VO_2_ kinetics, muscle oxygenation) and psychological state (“the operator”; e.g., motivation, perceived exertion, risk tolerance), prior experience (e.g., variable training conditions [101]), and tactical objectives. This integrated system is introduced in this review as the mind‐muscle‐environment interaction.

Through this lens, pacing is not the execution of a predetermined plan but an ongoing, real‐time decision‐making process that integrates sensory feedback, motor planning, environmental stimuli, and motivational priorities [17]. This becomes particularly relevant for the more complex competition formats involving head‐to‐head competition [19], as is common in the mass‐start events in cross‐country skiing and biathlon, as well as short‐track speed skating, where the impact of different competitive environments on pacing and performance has been reported [66]. Additionally, it allows room for a better understanding of how athletes cope with undulating terrains and changing weather circumstances, relevant in skiing events.

**FIGURE 1:**
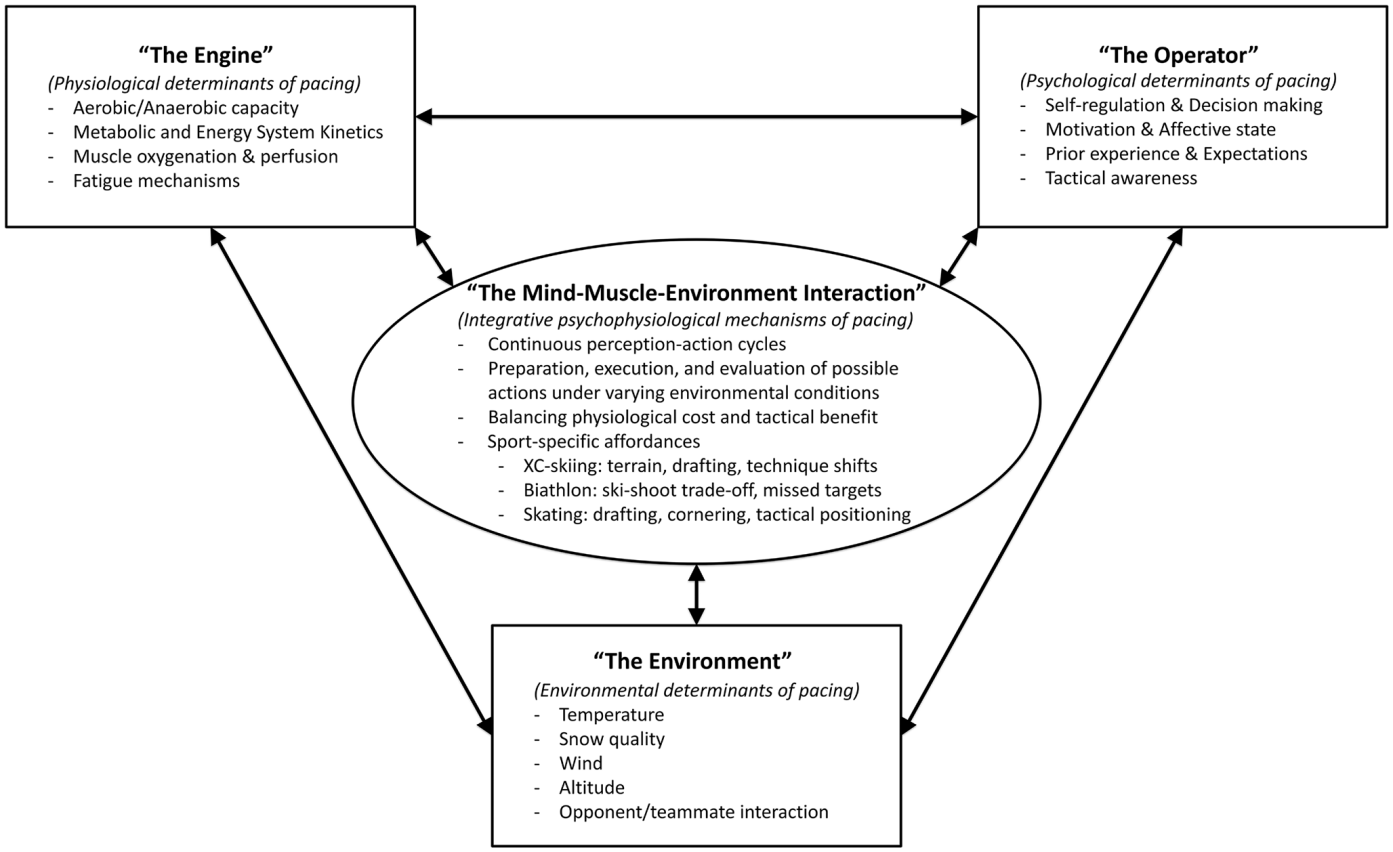
A unified framework for optimal pacing in Olympic winter endurance sports: Cross‐country skiing, Biathlon, and skating.

A multilayered control structure enables athletes to maintain adherence to an overarching race plan while remaining tactically flexible in response to shifting environmental and competitive cues. By embedding the “engine” within the “operator” in a real‐time perception‐action cycle, the affordance competition framework offers a coherent foundation for understanding and optimizing pacing strategies in preparation for Milano–Cortina 2026 Winter Olympics (Figure 1).

## 4 Winter Sport‐Specific Pacing Profiles: Cross‐Country Skiing, Biathlon, and Speed Skating

### 4.1 Cross‐Country Skiing

**FIGURE 2:**
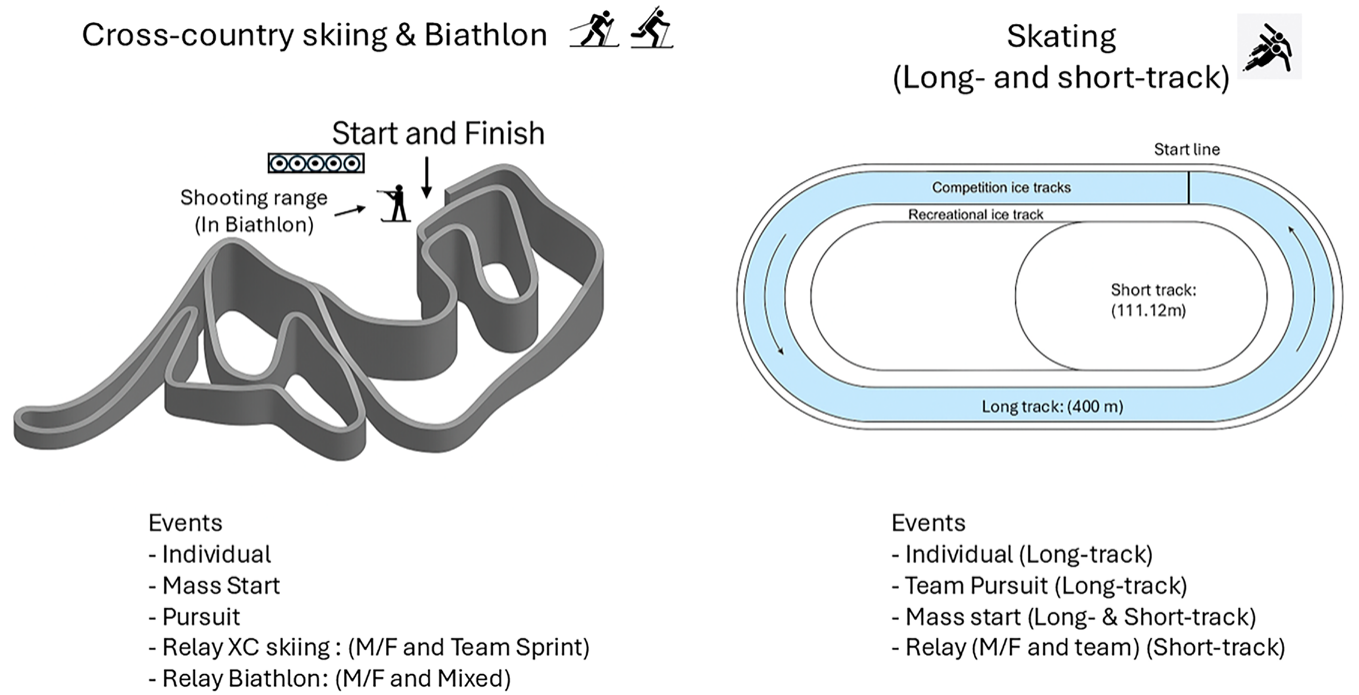
Schematic overview of competition formats and track profiles in cross‐country skiing, biathlon, and speed skating (long‐ and short‐track).

Cross‐country skiing has been an Olympic event since the first Winter Games in 1924 [7]. In modern times, the sport has undergone a significant transformation with the introduction of the skating technique and mass‐start and sprint formats, which have reappraised the physiological and tactical demands on the athletes (Figure 2) [38]. Current Olympic competitions cover distances from 1.3 to 50 km, with race durations ranging from approximately 3 min to over 2 h [38]. Although race courses have roughly equal sections of uphill, flat, and downhill terrain, more than half of the actual racing time is spent skiing uphill [7], making uphill skiing capacity a critical determinant of performance [38].

This is mirrored by a very high “engine” where Olympic skiers typically achieve VO_2_max values of ≈80–90 mL/kg/min (men), and ≈70–80 mL/kg/min (women) [38, 43, 102]. The technical demands are also considerable, as skiers change sub‐technique 15–25 times per kilometer in response to variable terrain, with each sub‐technique requiring different contributions from the upper and lower body, while speeds ranging from 5 to 70 km/h place additional pressure on the “operator” [7]. During a 1.5‐km sprint, for example, skiers may execute around 30 technique transitions, whereas longer races may involve several hundred, a stark contrast to the fewer transitions observed in other Olympic endurance sports with multiple techniques (such as swimming (medley) or triathlon) [20].

The marked differences in duration, terrain, and technical demands across cross‐country skiing events put more pressure on the athlete and make pacing a critical determinant of success [29, 103]. Analyses of elite‐level competitions consistently reveal that a positive pacing pattern, characterized by a gradual decline in velocity, is typically adopted, regardless of distance, technique, or sex [2, 21, 31]. However, substantial within‐lap variability is observed due to the undulating terrain and frequent transitions between sub‐techniques [6]. Skiers who effectively control their start pace during the first 2–3 min, along with possessing high aerobic capacity, strong technical skills, efficient recovery on downhills, and tolerance to repeated high‐intensity efforts, are better able to sustain an even pace throughout the race [34, 57].

Pacing profiles also have been found to differ according to event format. In individual time trials, athletes generally adopt a positive pacing strategy with lap‐to‐lap speed reductions of 2%–12% [6], while in mass‐start events, pacing becomes more stochastic due to tactical positioning, drafting opportunities, and responses to opponents' surges [6, 45]. These races often begin with a fast start to secure optimal positioning before narrowing sections, followed by variable intensities mid‐race and a decisive end‐spurt, yielding “J‐shaped” or “U‐shaped” pacing profiles [45]. In sprint skiing, success is strongly associated with anaerobic energy production, which accounts for a substantial portion of performance variability [21, 38, 104]. Sprint races often require a fast start to establish position, the ability to sustain high speeds in critical uphill sections, and the capacity to produce a fast finish sprint [30, 46].

Across formats, uphill skiing capacity is the strongest predictor of overall performance, accounting for ~50% of race time and showing an especially strong association in women [48, 52]. Faster skiers not only generate higher propulsive power but also employ more efficient sub‐techniques, such as double poling in classic style and gear 3 skating in freestyle, on given gradients [46, 52, 105]. In both sprint and distance races, longer cycle lengths, rather than higher cycle rates, are a primary determinant of skiing velocity across terrains [2]. The intermittent nature of cross‐country skiing, with terrain segments often lasting between 10 and 35 s, necessitates sophisticated “micro‐pacing strategies” and continuous speed regulation, particularly in key uphill sections where time gains or losses are magnified [6, 30, 45, 51]. Finally, environmental (e.g., cold and altitude) and equipment‐related factors (e.g., snow conditions, ski‐snow friction, and waxing quality) can substantially influence pacing, with deteriorating glide over time potentially contributing to the prevalence of positive pacing patterns in competition [2, 3].

Taken together, cross‐country skiing is characterized by a highly variable intensity profile in which the physiological “engine” must sustain exceptional aerobic power, repeatedly generating high outputs on climbs while recovering on descents [38]. At the same time, the psychological “operator” must anticipate terrain changes, regulate pacing across stochastic intervals, and adapt tactics in head‐to‐head racing. The integration of these roles becomes especially apparent in sprint and mass‐start formats, where drafting, surging, and positioning interact dynamically with terrain and snow conditions. Thus, pacing in cross‐country skiing can be framed within the affordance competition model: multiple opportunities for action are provided by the environment and the fellow competitors, such as attacking an uphill, conserving energy in a draft, or surging toward the finish, and these arise and compete for decision in real time. The skier's success ultimately depends on aligning physiological capacity with tactical intelligence in this dynamic environment.

### 4.2 Biathlon

Biathlon uniquely combines high‐intensity cross‐country skiing with precision rifle shooting [8]. Athletes ski using the skating technique while carrying a rifle (minimum 3.5 kg), which imposes substantial physiological demands comparable to elite cross‐country skiing [8] and simultaneously requires fine motor control for rapid and accurate shooting under considerable psychological pressure [106]. Race formats range from short‐sprint races (~7.5–10 km; ~20 min) to longer individual races (~15–20 km; ~45 min) and mass‐start, relays and pursuit events with four shooting bouts (Figure 2) [8]. Of the eleven Olympic biathlon events, seven now involve either mass‐start or pursuit formats, increasing tactical complexity and often deciding the podium during the final shooting bout or concluding ski sprint [8, 55]. Physiologically, elite biathletes possess high maximal aerobic power (VO_2_max > 80 and > 65 mL/kg/min in men and women, respectively) [102], with approximately 50% of total racing time spent skiing uphill, where most inter‐athlete performance variance occurs [8]. Shooting performance under fatigue demands advanced psychophysiological control, as athletes must stabilize their breath aim and fire five shots in ~25–30 s immediately following intense exertion [107]. While time spent on the shooting range contributes only a small fraction to total time, shooting accuracy is paramount; to be competitive for an Olympic medal, biathletes must achieve near‐perfect shooting accuracy, incurring no more than one or two misses in most events [56, 58].

Pacing in biathlon is a dual‐optimization problem that requires maintaining high skiing speeds while preserving the stability and fine motor control necessary for accurate shooting [108]. Physiologically, the “engine” is taxed by repeated stochastic fluctuations in skiing intensity across undulating terrain, elevated heart rate, and ventilatory strain. Psychologically, the “operator” must simultaneously manage arousal, attentional focus, and decision‐making under time pressure, particularly during shooting bouts where even minor decrements in fine motor control can result in large performance costs. While advances in track preparation, ski equipment, and physical conditioning have improved skiing speeds, shooting performance has not shown equivalent gains, amplifying the need for sophisticated pacing strategies [108]. Across competition formats, sprint, pursuit, individual, and mass‐start, athletes must balance high‐intensity skiing with the fine motor control required for accurate shooting, where penalties for missed targets (either time or penalty loops) can substantially alter the optimal pacing profile [8, 108]. Performance analyses of International Biathlon Union (IBU) World Cup events show that skiing speed explains ca. 50%–60% of performance variance in sprint races, whereas skiing and shooting contribute more equally to overall outcomes in individual competitions [58, 59]. Pacing profiles are most commonly “J‐shaped” or “U‐shaped,” with a fast opening lap before prone shooting, a slower middle lap, and a faster final lap toward the finish [6, 57]. However, recent experimental evidence suggests that moderating the intensity of the first lap (including the first demanding uphill), creating a more even pacing distribution, can improve total skiing time by ~1.5% without impairing shooting performance, while reducing perceived exertion early in the race [57]. Observational data indicate that top performers avoid overly aggressive starts, sustain relatively higher speeds in the latter laps, and sometimes decelerate slightly before standing shooting to enhance postural stability and accuracy [55, 57].

Beyond lap‐to‐lap regulation, micro‐pacing within each lap is important for overall performance. While high skiing intensity can yield time gains, excessive physiological strain, reflected in elevated heart rate and ventilation, may impair shooting accuracy by increasing rifle sway, tremor, and reducing postural stability [106, 108]. Head‐to‐head race formats, combined with penalties for missed shots, further amplify this interactive and stochastic nature of pacing. Athletes must decide not only how to distribute their energy over various terrain, but also how to dynamically throttle their “engine” in the approach to the range to stabilize the rifle and maintain accuracy. For example, studies show that slight reductions in skiing intensity and careful control of breathing before shooting improve accuracy despite the physiological drive to sustain high speed on the track. Here, in other words, the “operator” exercises restraint, modulating output from the “engine” to preserve precision. Elite biathletes therefore employ deliberate pacing adjustments or brief intensity reductions before shooting bouts, particularly in the prone position, to optimize hit rates [106]. For example, a recent study by Losnegard et al. [109] showed that highly trained biathletes systematically reduce skiing intensity before shooting, while maintaining a similar pacing pattern to cross‐country skiing during the rest of the race. Heart rate and perceived exertion did not differ substantially between the two disciplines, indicating that overall effort was comparable. Athletes reported adjusting their pacing based on in‐race experiences, and their strategies aligned more closely with prerace plans in biathlon than in cross‐country skiing. They relied primarily on sensations of leg fatigue rather than objective feedback such as time or heart rate. These findings highlight the complexity of pacing in biathlon, where athletes must balance high‐intensity skiing with the precision required for accurate shooting.

Moreover, faster athletes achieve higher instantaneous speeds in key course segments, including both steep uphills and technical downhills, with the greatest time gains often occurring in transitions after sharp turns or in complex downhill sections where stability and control are critical [61]. Carrying the rifle increases upper‐body loading and alters skiing mechanics, amplifying the relative importance of uphill sections for performance differentiation [61]. Technological advances such as GNSS tracking have enabled terrain‐specific performance analyses, consistently showing that top performers minimize speed loss in technical descents while sustaining higher velocities on steep climbs [6].

In summary, pacing in biathlon can be understood as the continuous negotiation between the physiological “engine” and the psychological “operator.” The “engine” delivers the power needed to ski fast across undulating terrain, while the “operator” must strategically regulate this output to preserve the fine motor control essential for accurate shooting. Within the framework of the affordance competition model, these decisions unfold as athletes experience competing opportunities for action, whether to attack an uphill, conserve energy before shooting, or respond to an opponent's surge. Ultimately, success in biathlon depends on who can most effectively harmonize these roles, knowing when to push the “engine” to its limits and when to ease off to maintain fine control.

### 4.3 Long and Short‐Track Speed Skating

Speed skating has featured at every Olympic Winter Games since 1924, with its modern rules governed by the International Skating Union since 1893. Today's Olympic long‐track speed skating program includes sprints (500 m and 1000 m), a middle‐distance event (1500 m), long‐distance races (3000 m, 5000 m, and 10 000 m), the mass‐start and the team pursuit (relay) (Figure 2) [67]. The short‐track speed skating events take place in heats on individual distances of 500, 1000, and 1500 m and the 2000, 3000 and 5000 m relays. Success depends on generating high mechanical power while minimizing frictional and aerodynamic losses. To reduce aerodynamic drag, elite skaters maintain an extreme crouched position that demands large isometric loads on the hip and knee extensors. This posture requires a small pre‐extension knee angle followed by rapid knee extension during the push‐off to maximize power [9, 110]. Physiologically, the deep crouched position also restricts muscle blood flow, which impairs muscle oxygenation [11] and contributes to elevated blood lactate concentrations across all Olympic distances [111]. Consequently, while both aerobic and anaerobic energy systems are critical, their relative importance and development can differ from other endurance sports. Although longer distances are predominantly aerobic [74], world‐class skaters often exhibit lower VO_2_max values than elite skiers or runners, and VO_2_max alone does not discriminate among medal contenders [10]. In short‐track, tactical aspects become increasingly important as competitions take place in heats with multiple opponents competing against each other per race [64, 70].

Pacing strategies in speed skating are closely shaped by the physiological, biomechanical, and tactical demands inherent to different race formats and distances. Across long‐track individual time trials, skaters typically adopt a positive pacing strategy, marked by an initial acceleration followed by a progressive decline in velocity. This pattern has been consistently documented in events ranging from 3000 to 10 000 m [67], and reflects the need to balance early momentum with the management of fatigue and technical precision over longer durations [15]. The 1500 m event presents a particularly complex pacing challenge, requiring athletes to optimize the trade‐off between a high early velocity and the preservation of skating technique throughout the race. Although modeling studies suggest a fast‐start strategy could be energetically optimal [63], empirical findings indicate that elite skaters often self‐select a more conservative initial pace, likely to reduce neuromuscular fatigue and mitigate biomechanical deterioration such as elevated trunk angles and compromised aerodynamic efficiency in the final laps [10, 72]. For shorter sprint distances (500–1000 m), performance is underpinned by a fast‐start or all‐out strategy that capitalizes on anaerobic capacity. Athletes who can attain and sustain high velocity early in the race without excessive pacing variability are more likely to achieve superior outcomes [69, 75]. However, in distances over 1000 m, skaters often moderate the first lap to avoid premature fatigue and maintain speed through the latter stages [69]. In contrast, the mass‐start event, which combines endurance with pack‐style racing, requires a hybrid pacing strategy where athletes adjust their effort around strategic laps (typically laps 4, 8, 12, and 16) to maximize point accumulation rather than minimize time [71]. In this format, positioning within the peloton, timing of sprint surges, and drafting play a greater role than consistent speed output [73]. Also in the team pursuit, drafting has been mentioned as a crucial, yet under‐understood, skill relevant to successful performance [73].

Short‐track racing introduces additional tactical complexity. Athletes must continually adjust their pace based on opponent behavior, track position, and race phase. Data from over 30 000 elite races show that pacing is significantly affected by contextual factors such as the number of competitors, race round, and the opportunity for time‐based qualification [66]. Tactical positioning, particularly early in the race, is critical, with skaters adjusting their strategy based on evolving interactions with competitors [64].

The mechanical context of long track speed skating also influences pacing decisions. Compared to other winter endurance sports, skating is conducted in a mechanically predictable environment, in a time‐trial setting on a flat, indoor 400 or 111.12‐m oval (speed skating and short track, respectively), which simplifies external resistance to three components: ice friction, aerodynamic drag, and centripetal force. This allows for accurate modeling of power output and optimal pacing trajectories [62, 74]. However, biomechanical constraints, such as the crouched posture adopted to minimize drag, impose physiological challenges, including reduced muscle perfusion and asymmetric oxygenation, particularly in the right leg during turns [11]. These physiological demands interact with choices regarding optimal pacing strategy. For instance, excessively high pace in the early segments of the race may lead to technical breakdown and reduced efficiency, especially during the latter stages of races, where muscle oxygenation is already compromised [10, 72]. These effects are particularly pronounced in short‐track and mass‐start events, where repeated cornering and tactical maneuvers outperforming opponents further amplify muscular strain and fatigue.

In speed skating, the biomechanical demands of the crouched position and powerful lateral push‐offs impose heavy isometric loads that restrict blood flow and accelerate fatigue [9, 10]. The “engine” must cope with high metabolic stress and asymmetric muscular oxygenation, particularly in short track where cornering is frequent [11]. Yet performance cannot be explained by physiology alone: the “operator” plays a central role in interpreting sensations of fatigue, managing perceived exertion, and making split‐second tactical decisions under head‐to‐head pressure. In long track skating, the engine's output needs to be distributed evenly, balancing explosive starts with technical efficiency across laps. In mass‐start or short‐track racing, athletes are constantly challenged by opponents' moves, drafting opportunities, or lane positions to alter pacing and a multitude of factors determine whether surges are sustainable or whether early aggression will lead to collapse in the closing laps, and the environment plays a significant role here. Unlike cross‐country skiing and biathlon, where high speeds are intermittent, the sustained high velocities in speed skating mean that reduced air density at moderate altitudes (e.g., Calgary, Salt Lake City) provides a significant performance advantage by lowering aerodynamic drag [62]. This creates a distinct strategic scenario where pacing decisions are not only about managing internal fatigue, but also about capitalizing on the known physical advantages of a specific environment.

In summary, pacing in speed skating reflects the constant negotiation between internal and external factors, and a large role for the environment in the form of opponents or teammates. A high‐power output must be generated despite large metabolic strain, restricted muscle oxygenation, and biomechanical constraints, while sensations of fatigue, opponent movements, and positional opportunities need to be incorporated to decide when to hold back and when to surge. Within the affordance competition model, these decisions represent a real‐time selection among competing opportunities for action, whether to conserve energy in the pack, exploit drafting on the straights, or launch a decisive sprint out of the final corner. The most successful skaters are those who can adapt to the demands of each moment, converting physiological power into well‐timed moves that preserve technique, exploit drafting, and capitalize on race dynamics across formats ranging from solo time trials to the tactical chaos of mass‐start and short‐track events.

### 4.4 A Unified Framework for Optimal Pacing

While classical pacing models, including the central governor and teleoanticipation [82, 83], and the psychobiological model [84] each provide valuable insights into the regulation of exercise intensity, they do not fully account for the adaptive, real‐time decision‐making seen in elite winter endurance sports. These sports, cross‐country skiing, biathlon, and speed skating, present athletes with highly dynamic environments, unpredictable competitor behavior, and complex equipment–athlete‐environment interactions that necessitate constant recalibration of effort. As mentioned before, the affordance competition hypothesis [18] offers a framework for integrating all these crucial determinants of pacing [16, 18, 19] in Olympic winter sports athletes.

In cross‐country skiing, this manifests in the stochastic interval profile typical of elite performance [2, 6], where terrain changes, sub‐technique transitions, and opponent surges continuously reshape the affordance landscape. Athletes must weigh the opportunity to attack on a climb against the metabolic cost and the need for recovery on subsequent descents, adjusting their pacing template through adaptive feedforward‐feedback loops [16, 18, 19]. In biathlon, the affordance space is further complicated by the dual‐optimization problem of maximizing ski speed while preserving the fine motor control required for accurate shooting [59, 108]. Here, decision‐making integrates the trade‐off between high‐intensity skiing, often decisive on uphills [61] and deliberate preshooting decelerations to stabilize aim [56]. The affordance competition model accounts for these micro‐adjustments, recognizing that athletes often downregulate effort not because of energy system depletion alone, but to optimize task‐specific precision under fatigue. In speed skating, particularly in short‐track and mass‐start formats, the competition between affordances is heavily shaped by drafting opportunities, pack dynamics, and lap‐specific tactical goals [17, 64]. Even in individual time trials, where the environment is more stable, pacing is constrained by biomechanical and physiological factors such as reduced muscle perfusion in the crouched position [11] and the aerodynamic cost of velocity maintenance [62].

The unified pacing model explains how skaters modulate effort to manage neuromuscular fatigue while exploiting race‐specific opportunities, such as timing the end‐spurt to maximize speed without incurring early technical breakdown. Also, it was found that the faster start, which is usually seen when competing against faster opponents, as established in cycling [112] diminished in a fatigued state [12, 13], showing that internal states such as fatigue impact pacing decisions evoked by the environmental factors, in this case, the opponents. Hereby establishing in a lab‐controlled situation that interrelations between fatigue, environmental stimuli, and pacing are playing a role in the final competitive outcome.

From a practical perspective, the unified pacing model underscores the importance of training athletes not only to develop aerobic and anaerobic capacity, but also to enhance perceptual‐cognitive skills: recognizing actionable race opportunities, interpreting internal cues in context, and recalibrating effort distribution under pressure. For Milano–Cortina 2026, athletes who can fluidly navigate this competition of affordances, balancing what is possible, what is physiologically sustainable, and what is tactically optimal, are most likely to translate capacity into podium performance.

This framework views pacing not as a rigid, preprogrammed execution of a race plan, but as an emergent behavior arising from the dynamic competition between:

– What is possible? The action opportunities (affordances) are defined by environmental conditions, terrain, opponents, and equipment.
– What is felt? The athlete's physiological and psychological status, including physical capacity, mood, and fatigue resistance.
– What is known? Prior experience, technical skill, tactical knowledge, and awareness of the task endpoint. To improve recognition and decision‐making regarding affordances, exposure to variable training conditions is essential.
– What happens in the environment? Over time, skilled performers learn to identify and prioritize affordances that maximize performance potential while managing physiological limits. In cross‐country skiing, this may involve micro‐adjustments to sub‐technique choice and power application to exploit terrain changes without inducing premature fatigue. In biathlon, learning to pace includes mastering the trade‐off between skiing intensity and shooting precision, such as modulating speed on the approach to the range to optimize stability under pressure. In speed skating, particularly in mass‐start or short‐track formats, it requires the ability to anticipate drafting opportunities, position effectively in the pack, and time decisive accelerations while maintaining technical efficiency in a crouched position that imposes unique physiological constraints.

### 4.5 Learning How to Pace

The ability to pace effectively is not an innate talent but a complex, learned skill that develops through years of deliberate practice, competitive exposure, and reflective refinement. Younger athletes typically display a more positive pacing pattern, characterized by relatively faster starts and greater deceleration than older and more experienced athletes [50]. To be able to understand the learning of the complex skill of pacing, starting from the initial work on teleoanticipation [82] and the performance template [88], there is another model that is highly applicable to Olympic winter endurance sports, and that is the model of self‐regulation of learning [113] applied to pacing by Elferink‐Gemser and Hettinga 2017 [81].

Crucial here is the Self‐Regulation Cycle, through which athletes construct and progressively optimize their performance template via a continuous loop of planning, monitoring, evaluating, and reflecting [81, 114]. This iterative process links prerace intentions with in‐race adaptations, allowing athletes to integrate both internal cues and external information to fine‐tune effort distribution. Self‐regulatory capacity matures with experience, but it can be systematically trained. Structured exposure to varied competitive scenarios, ranging from low‐stakes simulation races to high‐pressure championship events, broadens the athlete's repertoire of viable pacing responses.

Coupling subjective signals such as RPE with objective performance metrics such as split times, power output, heart rate, and lactate data enhances the calibration between perception and output, improving decision‐making accuracy under pressure [17, 81]. This calibration is particularly critical in winter endurance sports, where environmental factors (snow conditions, temperature, wind, and altitude) and tactical variables (opponent surges, drafting opportunities) can rapidly alter the optimal pacing strategy. Cognitive functions involved in the monitoring and adaptation of the distribution of effort during exercise underpin the development of pacing behavior, with a role for feedback from the (social) environment [115].

A potential key component of this learning process is the Internal Clock, a largely subconscious timing mechanism that provides scalar information about the remaining distance or duration of an event [79]. This temporal framework enables the brain to scale effort appropriately to the endpoint, facilitating a proportional increase in work rate as the finish line approaches. An additional complexity that has remained underexplored is the fact that exercise and exercise intensity influence the perception of time [116]. While much of this timing regulation operates below conscious awareness, heightened task strain can draw it into conscious focus, where it is integrated with interoceptive feedback (e.g., breathing discomfort and muscular fatigue) and perceived exertion to guide moment‐to‐moment pacing decisions [76].

Learning how to pace includes integrating perceptual‐cognitive skill acquisition with physiological training under race‐specific conditions [114]. This integration allows athletes to interpret internal signals in context, adjust effort distribution to the demands of both the environment and competition, and execute a flexible yet goal‐driven race strategy. For Olympic endurance winter sports, developing these skills represents a decisive step toward converting physical capacity into championship performance.

## 5 The Milano‐Cortina Playbook: Translating Science Into Gold Medal Strategies

### 5.1 Practical Applications: A Phased Pacing Playbook

Translating the above into practice requires integrating the athlete's “engine” (capacity) with the “operator” (decision‐making), allowing room for environmental challenges. The self‐regulation model applied to pacing, using self‐regulation to build robust templates with a role for teleoanticipation [81, 115, 117], is of interest when we want to understand how coaches and athletes can learn and develop the complex skill of pacing. The affordance competition hypothesis [16, 18, 19] applied to pacing is of particular interest to guide real‐time action selection when conditions change.

### 5.2 Preseason Diagnostics and Planning

From a self‐regulation perspective, athletes learn to master a skill through iterations of the self‐regulation cycle (plan‐monitor‐evaluate‐reflect) under race‐specific conditions [81, 115, 117]. Training should systematically expand pacing repertoires (even, positive, negative, and variable) across distances and terrains, using both interval and continuous formats, and include test races on the actual competition track when possible, as such simulations, combined with GPS analysis, can substantially improve pacing calibration and performance [6], and include race simulations to capture competitive intensity. Sport‐specific movements (skiing, skating, or combined ski‐shoot tasks in biathlon) are essential to ensure ecological validity, accommodating sport‐specific differences. Targeted feedback (e.g., split or lap information) helps refine calibration and enhance control of start pace in the first 2–3 min, reducing the tendency toward overly aggressive starts, particularly in youth [34, 114, 118].

From an ecological affordance competition perspective, training should also develop athletes' ability to detect and exploit the affordances of terrain, opponents, and bodily state [17]. This requires practising under variable race‐like conditions, where interval intensity, tactical scenarios, and terrain changes continuously invite different actions [101]. Such exposure broadens the set of prepared responses and tunes biasing inputs (goals, risks, and interoceptive state), enabling faster and more adaptive resolution of competition among feasible actions without relying on step‐by‐step calculations [17, 19]. This process involves a continuous and simultaneous interaction between environmental stimuli and an athlete's action capabilities [66].

### 5.3 Competition‐Day Execution

On competition day, effective pacing requires balancing adherence to a preplanned strategy with the flexibility to adapt to dynamic race conditions. From a self‐regulation perspective, athletes use imagery, deliberate self‐talk, and attentional control to regulate arousal, manage discomfort, and sustain focus [81]. Dynamic self‐regulation integrates both objective (e.g., split times and heart rate) and subjective (e.g., RPE, breathing rhythm, and local muscle tension) cues to guide real‐time adjustments in effort [117]. Simultaneously, the affordance competition hypothesis frames pacing as a continuous process of action selection, where athletes perceive and evaluate emerging opportunities, such as terrain changes, opponent surges, or environmental shifts, and adjust their strategy accordingly [16, 17]. Fatigue and RPE act as biasing signals, while contextual rewards and risks shape which action “wins” and is executed. Successful pacing thus depends on integrating deliberate self‐regulation to develop an optimal strategic approach to pacing, with the more intuitive ability to optimize unanticipated affordance selection, which is particularly relevant under changing circumstances (related to terrain, climate, or competition, as is the case in winter sports).

### 5.4 Sport‐Specific Cues

While the underlying psychophysiological principles of pacing are shared across winter endurance sports, their application must be tailored to the unique environmental and tactical demands of each discipline. On race day, this means translating self‐regulatory control and affordance awareness into sport‐specific strategies.

In cross‐country skiing, athletes should exploit climbs, maintain technique during transitions, and maximize recovery on descents while adapting pacing to snow conditions, altitude, and opponent behavior [2, 6]. In biathlon, success depends on balancing ski speed with shooting precision by moderating intensity before the range, particularly before standing bouts, where postural stability and breath control are critical [8, 56]. Performance development, including the optimization of pacing strategies and tactical decision‐making, requires extensive specific training. A series of studies by Losnegard et al. [34, 57, 119] has shown that adopting a conservative start during the first 2–3 min of a race can substantially improve overall performance, further emphasizing the importance of even pacing. Since no external tools can be used during races to control speed due to the stochastic interval profile, the ability to self‐regulate effort emerges as a critical performance characteristic for reaching elite level. In this context, the specificity of practice becomes essential, and evaluating lap times or segment speeds, using Global Navigation Satellite System (GNSS) based measurements, offers a valuable method to plan and objectively assess competitions or training sessions. Such evaluations, combined with subjective measures like ratings of perceived exertion (RPE), can support individualized pacing strategies and provide meaningful feedback for both elite and developing athletes [119].

In speed skating, especially in mass‐start and short‐track events, athletes must optimize drafting and positioning, time decisive surges, and preserve technique under rising fatigue and declining muscle oxygenation is of importance [11, 66]. Ultimately, mastering pacing requires sport‐specific execution of general principles, integrating perception, physiology, and tactics in real time.

### 5.5 How Can Technology Help Athletes Improve Pacing?

Emerging technologies enable athletes to refine pacing control and decision‐making through precise feedback and simulation. GNSS and inertial measurement unit (IMU) data, combined with synchronized video analysis, allow “micro‐pacing” and terrain‐ or corner‐specific velocity optimization in cross‐country, biathlon and speed skating [6, 51, 61]. Manipulated feedback interventions, such as strategic deception of lap or distance information, can safely challenge athletes' perceived exertion limits and recalibrate their internal model of effort regulation [99]. Virtual‐reality or avatar‐based opponents add psychological realism, facilitating the practice of tactical decision‐making under uncertainty and competitive stress [116]. Integrating these tools within ecologically valid settings allows athletes to explore affordances of the performance environment while maintaining self‐regulatory control. Coaches should also monitor competitive impulses during submaximal training sessions, as technology‐mediated feedback may unintentionally provoke pacing responses that alter workload and recovery demands [120].

### 5.6 From Framework to Gold Medal Execution

The affordance competition hypothesis provides the decision‐making scaffold for this playbook: on race day, athletes continuously weigh what is possible (affordances shaped by terrain, opponents, and environment), what is felt (current physiological state), and what is known (experience, race plan, and endpoint) to select actions that maximize performance potential. Self‐regulation theory is useful to understand the learning‐related elements and develop guidelines for coaching and athletes to facilitate the learning process.

### 5.7 Limitations

This review has several limitations that should be acknowledged. First, although we aimed for a comprehensive synthesis of the psychophysiological determinants of pacing in Olympic winter endurance sports, our structured narrative approach does not provide the quantitative precision of a meta‐analysis. Moreover, much of the current literature is based on small sample sizes, predominantly involving male athletes at the national to world‐class level. As a result, the generalizability of findings to women athletes, youth, and para‐athletes remains uncertain.

Potential sex‐based differences in pacing behavior also warrant consideration. Evidence indicates that physiological capacity, tactical choices, and risk‐taking tendencies may differ between men and women, which could influence how specific components of the framework apply across sexes [31, 50, 71]. Future research should examine how these sex‐specific factors interact with environmental conditions and event formats to refine and validate the framework for broader athlete populations. Moreover, several of the included studies were conducted in controlled laboratory or simulated competition settings, which, while informative, may not fully capture the stochastic demands, tactical complexity, and environmental variability characteristic of real‐world competitions. A further limitation is the challenge of empirically validating a multi‐factor pacing framework in complex real‐world winter sport environments. Future studies should integrate advanced in‐race monitoring technologies (e.g., heart rate, continuous lactate sensing, NIRS), athlete–opponent interaction modeling, and longitudinal performance tracking across seasons. Such methods will support systematic testing, refinement, and eventual translation of the framework into practical tools for coaches and high‐performance practitioners. Lastly, psychophysiological models of pacing remain challenging to validate directly, as measurements of cognitive processes, perception of effort, and decision‐making under competitive stress are inherently indirect and context dependent.

### 5.8 Future Research Directions

Despite recent advances, important knowledge gaps remain. Future work should move beyond descriptive interpretations toward targeted hypothesis testing, explicitly exploring psychophysiological processes and their interaction with the competitive environment. With respect to the environmental interface, research should address pacing at altitude, where hypoxia may alter the balance between physical capacity and tactical decision‐making. This is timely, as future international competitions are increasingly likely to be held at moderate altitude, yet experimental studies are lacking. Likewise, mass‐start formats remain underexplored despite their growing prominence; here, the density of athlete–opponent interactions may fundamentally change pacing strategies compared with time‐trial formats.

Future research should also investigate how manipulating opponent behavior (e.g., surges and drafting opportunities) influences pacing flexibility and tactical decision‐making under pressure, in line with the affordance competition perspective. Finally, longitudinal research should test whether systematic self‐regulation training during youth accelerates the development of mature pacing strategies and reduces race‐to‐race variability in adulthood, a defining feature of the operator's psychophysiological maturation. Addressing these hypotheses with real‐time monitoring technologies (EEG, fNIRS, GNSS, and IMUs) in ecologically valid competition settings will provide the causal evidence needed to refine psychophysiological models of pacing.

## 6 Conclusion

In the pursuit of Olympic success at Milano–Cortina 2026, excellence in winter endurance sports will depend on more than the raw horsepower of the physiological “engine”. Environmental factors play an essential role in a variety of winter sports; therefore, it is important to comprehend human–environment interactions. Across cross‐country skiing, biathlon, and speed skating, performance emerges from the real‐time competition between affordances: opportunities to attack, to draft, to conserve energy, or to stabilize before shooting. To understand more about how to learn the complex skill of pacing, the self‐regulation model applied to pacing is of interest, which can provide guidance to coaches and athletes using the reflective cycle. Although this manuscript uses the upcoming Milano–Cortina 2026 Olympics as an example, the framework is not limited to a single Games. It offers a transferable scientific foundation for understanding and optimizing pacing in cross‐country skiing, biathlon, and skating. As winter sports evolve in format, technology, and environmental demands, the framework provides a consistent tool for integrating new evidence and guiding pacing strategies. Its relevance, therefore extends well beyond 2026 and supports long‐term progress in pacing research and applied practice.

### 6.1 Perspective

This review highlights that pacing in Olympic winter endurance sports emerges from dynamic mind‐muscle‐environment interactions rather than from isolated physiological or psychological mechanisms. Building on the affordance competition hypothesis, we propose pacing as a continuous decision‐making process in which athletes select and adjust their actions based on perceived affordances and changing internal states. The affordance competition hypothesis provides the neural mechanism through which motor and perceptual options are continuously selected and evaluated. This allows athletes to change their pacing, adapting to varying circumstances and multiple opponents, which is particularly relevant in speed skating, biathlon, and skiing events. In reviewing literature on these winter sports, it also comes forward that performance is limited by the athlete's psychophysiological state, linking effort regulation to both physiological and psychological control. To guide athletes in optimizing this interplay and enabling athletes to perceive and act upon affordances more efficiently in varying situations, the self‐regulation of learning model could provide a useful framework to help coaches teach their athletes the complex skill of pacing. Future research using in‐competition monitoring (e.g., GNSS, fNIRS, and IMU) should test how athletes perceive, interpret, and act upon dynamic affordances to optimize pacing. Such work may bridge theory and performance, guiding athlete preparation for Milano–Cortina 2026 and beyond.

## Funding

The authors have nothing to report.

## Conflicts of Interest

The authors declare no conflicts of interest.

## Data Availability

Data sharing is not applicable to this article as no datasets were generated or analyzed during this study.
